# Exploring the Properties and Indications of Chairside CAD/CAM Materials in Restorative Dentistry

**DOI:** 10.3390/jfb16020046

**Published:** 2025-02-01

**Authors:** Codruţa-Eliza Ille, Anca Jivănescu, Daniel Pop, Eniko Tunde Stoica, Razvan Flueras, Ioana-Cristina Talpoş-Niculescu, Raluca Mioara Cosoroabă, Ramona-Amina Popovici, Iustin Olariu

**Affiliations:** 1 Faculty of Dental Medicine, “Victor Babes” University of Medicine and Pharmacy, Revolutiei Ave. 1989, No. 9, 300580 Timișoara, Romania; ille.codruta@umft.ro (C.-E.I.); pop.daniel@umft.ro (D.P.); stoica.eniko@umft.ro (E.T.S.); ioana.talpos-niculescu@umft.ro (I.-C.T.-N.); cosoroaba.raluca@umft.ro (R.M.C.); ramona.popovici@umft.ro (R.-A.P.); 2 TADERP Research Center—Advanced and Digital Techniques for Endodontic, Restorative and Prosthetic Treatment, “Victor Babeș” University of Medicine and Pharmacy, Revolutiei Ave. 1989, No. 9, 300041 Timişoara, Romania; 3 Faculty of Dental Medicine, Vasile Goldiş Western University of Arad, 86 Liviu Rebreanu Street, 310414 Arad, Romania; razvanflueras@gmail.com (R.F.); olariu.iustin@uvvg.ro (I.O.)

**Keywords:** biomaterials, chairside CAD/CAM materials, prosthetic dentistry, minimally invasive dentistry

## Abstract

The present review provides an up-to-date overview of chairside CAD/CAM materials used in restorative dentistry, focusing on their classification, properties, and clinical applications. If CAD/CAM technology was only an aspiration in the past, a higher proportion of clinics are employing it nowadays. The market is overflowing with biomaterials, and these materials are constantly evolving, making it challenging for practitioners to choose the most appropriate one, especially in correlation with patients’ medical diseases. The evolution of CAD/CAM technology has revolutionized dental practice, enabling the efficient fabrication of high-quality restorations in a single appointment. The main categories of chairside CAD/CAM materials include feldspathic ceramics, leucite-reinforced ceramics, lithium disilicate, zirconia, hybrid ceramics, and acrylic resins. The mechanical, physical, and aesthetic properties of these materials are discussed, along with their advantages and limitations for different clinical scenarios. Factors influencing material selection, such as strength, aesthetics, and ease of use, are also assessed. Ultimately, the guiding principle of dentistry is minimally invasive treatment following the particularity of the clinical case to obtain the envisioned result. Correlating all these factors, a simple, up-to-date classification is required to begin an individualized treatment. By synthesizing current evidence, this comprehensive review aims to guide clinicians in selecting appropriate chairside CAD/CAM materials to achieve optimal functional and aesthetic outcomes in restorative procedures. The integration of digital workflows and continued development of novel materials promise to further enhance the capabilities of chairside CAD/CAM systems in modern dental practice.

## 1. Introduction

CAD/CAM (Computer-Aided Design/Computer-Aided Manufacturing) technology has revolutionized many fields, including dentistry. Broadly, CAD/CAM integrates computer technology into design and manufacturing processes, enabling greater precision, efficiency, and customization in product development. In dentistry, this technology has been increasingly adopted for the fabrication of dental restorations, including crowns, bridges, and dentures, significantly improving the quality and speed of dental care. One of the goals of the medical field is to improve quality of life, and thanks to advancements in dentistry and the introduction of intelligent dental products, this aim can now be more successfully attained. With the use of CAD/CAM technology, nearly the whole spectrum of fixed and removable prosthetic restorations on natural teeth or implants can now be developed, starting with basic crowns and inlays [1].

The first commercially available dental CAD/CAM system was CEREC, developed in the 1980s, marking a turning point in dental technology [2]. Some branches of this system have enabled the digital design and fabrication of dentures, and it has since evolved to include various applications such as orthodontics and removable prosthetics [3,4]. CAD/CAM technology enables the creation of highly aesthetic and functional restorations that closely resemble natural teeth, improving patient satisfaction and outcomes [5].

Significant progress in recent years has shown that CAD/CAM technology not only improves the accuracy of dental restorations but also reduces the time needed for procedures. For instance, incorporating intraoral scanners into CAD/CAM technology has been reported to reduce procedure time and increase patient comfort while maintaining a high level of accuracy [6].

The uniqueness of chairside systems is that the clinician can scan the tooth preparation intraorally and by selecting appropriate materials can fabricate the restorations and seat it within a single appointment [7]. Any CAD/CAM system requires these three steps: in-office digital impression or scanning to gather and store information about the oral environment (tooth preparation, neighboring teeth, and occlusive tooth shape); CAD to design the restoration and complete the process under-recognized dental standards, and CAM for manufacturing the restoration. When compared to more conventional techniques, these instruments offer several benefits for capturing intraoral structures, such as shorter clinical times, more patient comfort, the ability to assess surgical preparations, reduced risk of cross-infection and impression distortion, and endless storage of digital models [2,8,9].

For chairside restorations, there are several design programs available, including CEREC (Dentsply Sirona) and Plan CAD Easy (Planmeca). The idea behind same-day crowns, also known as same-day dentistry, is that the doctor chooses the case, prepares it, scans it intraorally, uses digital design, chooses the materials, mills it, posts it, and finishes it up to final cementation. The clinician must carefully examine and arrange the course of treatment for future restoration before creating the final digital design. An in-office software can be used to construct a variety of minimally invasive restorations, including veneers, inlays, onlays, crowns, tabletops, endo-crowns, and several single-unit restorations [10,11]. The digital processes for chairside oral rehabilitation imply that the architectural data generated by CAD software are to be placed onto a milling unit and transformed into milling strips for CAM processing. Integrating these data into milling units for CAM processing represents the intersection of design and production technologies. The number of milling axes serves as a key differentiator between machining devices, influencing the functionality, versatility, and complexity of the final product [2,9].

Among the general advantages of CAD/CAM technology are rapidity and simplicity of use, superior restoration quality, reduced labor, and time commitment. In addition to that, all scans are computer-storable, impressions are accurate, patients do not need to take a physical impression or visualize the occlusion, accurate restorations are created on digital models, there are no layering or baking errors or casting/soldering errors, it is cost-effective, cross-contamination control is possible, and in-office systems save time and require fewer visits. Despite the advantages, implementing CAD/CAM technology is not without its challenges. The disadvantages are the large change in workflow, which can be a barrier for some dental professionals [12].

CAD/CAM chairside ceramic materials can be divided into four groups based on their composition and functions, such as feldspathic ceramics and leucite-reinforced ceramic blocks, lithium disilicate and zirconia-reinforced lithium silicate blocks, zirconium oxide, hybrid ceramics or resin matrix ceramic materials, as well as acrylic resin. For instance, feldspathic ceramics (such as CEREC Blocs from Dentsply Sirona and Vitalocs Mark II from Vita Zahnfabrik) are examples of the first line of chairside CAD/CAM materials. These materials were first made accessible for commercial use before 1990. They were the most researched materials from a lifespan perspective and held a dominant position in the market until the 2000s. Feldspathic ceramics are among the most transparent and aesthetically beautiful ceramic materials, with a glassy phase preponderance of 55–70% [13,14]. Since feldspathic ceramics (Vita Mark I, Vita Zahnfabrick, Bad Sackingen, Germany) were the only materials accessible at first, the need for improved mechanical qualities led to the development of other materials that might increase the indicators of CAD/CAM restorations (onlays, crowns). Zirconia, lithium disilicate glass ceramics, and leucite are a few examples of these microstructures. To ensure quick milling, some of the previously described components may be present in a pre-crystallized stage. However, to obtain the final shade and the appropriate mechanical strength, a post-milling crystallization is required [15,16]. Hybrid ceramic materials and CAD/CAM resin composites have recently been made available. Benefits are easy intraoral repair with light-cured restoratives and a faster production rate, as firing is not needed [17].

In the field of dental materials, the evolution of ceramic materials has significantly impacted restorative dentistry. Initially, lithium disilicate (IPS e.max CAD Ivoclar Vivadent) was introduced in 2006, recognized for its superior mechanical strength and aesthetic outcomes, with a bending resistance exceeding 350 MPa, which positioned it as a leading material in fixed prostheses. Its ability to bond effectively to tooth structures further enhances its clinical utility, making it a preferred choice among dental professionals [14,18,19]. Following the success of lithium disilicate, monolithic zirconia was introduced, which offered enhanced strength and durability, particularly in posterior restorations. This material became popular due to its high fracture resistance and suitability for high-stress applications. Subsequently, in 2016, zirconia-reinforced lithium silicate (ZLS) blocks emerged as a significant advancement in dental materials. These blocks combine the aesthetic benefits of lithium disilicate with the mechanical strength of zirconia, resulting in a material that not only maintains the translucency and color stability characteristic of lithium disilicate but also enhances the overall strength and fracture resistance. The incorporation of approximately 10% zirconia into the lithium silicate matrix leads to a denser crystalline structure, improving the optical properties and translucency of the final product [14,15,20,21]. The aesthetic qualities of ZLS, including its translucency and color stability, are critical factors that position it as a first-choice material in restorative dentistry. Studies have shown that ZLS exhibits lower light reflection compared to lithium disilicate, indicating better optical properties that are essential for achieving natural-looking restorations. Furthermore, ZLS has demonstrated superior color stability compared to other materials, which is vital for long-term aesthetic outcomes in dental restorations [14,22,23].

In the field of dental implants, orthopedics, and advanced ceramics, zirconium oxide is recognized for its excellent mechanical and antibacterial properties and biocompatibility, making it a material of election. Baus-Dominguez et al. have shown that by modifying the surface of zirconium oxide, its biological properties can be enhanced, as it leads to better sealing of soft tissues and reduces bacterial colonization, which is essential for the longevity of implants [24]. The potent bactericidal activity of zirconium oxide is also supported by Shannon et al. [25], who reported that zirconium oxide efficiently inhibited various bacterial species, an activity essential regarding its use in clinical settings where infection control is necessary. Zirconium oxide also has potential in restorative dentistry, by combining it with different cements (e.g., calcium silicate cement), leading to the improved compressive strength of the final product [26]. The attributes listed above make zirconium oxide a valuable material in biomedical applications, with ongoing studies aimed at optimizing its properties for various other uses.

Hybrid ceramics have attracted the attention of dentists for their ability to mimic the properties of natural teeth, offering increased durability and aesthetics, as they combine the advantages of both ceramics and composite resins. Their structural composition, which includes a polymer matrix reinforced with inorganic filler particles, allows for improved flexibility and fracture resistance compared to traditional ceramics, making these materials suitable for various dental restorations, including crowns and veneers [27,28]. Since the flexural strength of these materials is comparable to that of natural teeth, because of the resin matrix, these ceramic materials can be successfully applied in load-bearing areas [29,30]. The performance of resin matrix ceramics is influenced by various factors, such as their porous structure, which leads to internal discolorations that are not easily removed by polishing; therefore, it is extremely important to carefully choose the material and the processing techniques [31,32]. In addition, surface modifications, such as laser texturing, can significantly increase the bond strength of resin matrix ceramics, which is essential for the longevity of dental restorations [33].

Acrylic resins, in particular polymethyl methacrylate (PMMA), are widely used due to their favorable mechanical properties, aesthetic qualities, and biocompatibility. But certain challenges such as bond strength, surface roughness, and color stability continue to be the focus of researchers. It has been shown that improving the bond strength between acrylic resins and other materials can be achieved by the application of adhesion promoters [34,35]. Surface modification techniques (e.g., micromechanical treatments) increase the longevity and performance of acrylic resins in the clinical setting [36,37]. The incorporation of nanofillers and other reinforcements improves the mechanical properties of acrylic resins, resulting in stronger and more durable materials [38,39]. For aesthetic applications, the color stability and surface characteristics of acrylic resins are important issues. It has been shown that exposure to various substances can adversely affect the surface roughness and color stability of acrylic resins. Soaking acrylic resins in certain solutions can lead to hydrolysis and the formation of cracks, which compromise the integrity of the material [40,41], and the polishing protocols applied to bis-acryl-based materials influence their wear resistance and surface smoothness, which affects the maintenance of their aesthetic quality over time [42].

Although advances in CAD/CAM technology have revolutionized the fabrication of dental restorations, allowing for the production of high-quality, durable, and esthetic restorations in a single appointment, the selection of appropriate materials for this chairside CAD/CAM technology remains an important consideration, due to factors such as mechanical properties, aesthetic outcomes, and clinical efficiency. Selecting the most appropriate material for chairside CAD/CAM technology has significant implications for both patient satisfaction and clinical workflow. After analyzing the material properties, clinicians should choose the systems that are designed to give the patient the best clinical result in terms of appearance, functionality, longevity, and compatibility with the natural tissues around them. According to an analysis of laboratory and clinical data, machined restorations are a dependable and aesthetically pleasing substitute that could yield better results than traditional fabrication processes.

Therefore, in the context of the above, this literature review aims to classify and characterize the most suitable materials for chairside CAD/CAM technology, materials that are resistant and meet the functional requirements of the oral environment; have a natural appearance; have excellent color stability, translucency, mechanical properties (including flexural strength and impact resistance), and aesthetic results; and have the longest possible life span.

### 1.1. A Brief History of Chairside CAD/CAM Technology

Over the past decades, it seems that the evolution of CAD/CAM (Computer-Aided Design/Computer-Aided Manufacturing) materials in dentistry has significantly transformed dental restorative practices. Although CAD/CAM technology may seem like a more modern choice in the dental restorative tool kit, it was first introduced in the 60s and 70s, mainly for industrial applications (automotive and aeronautics). A decade later, in the 1980s, this technology made its way into the dental field, with systems such as CEREC (Sirona) and Procera (Nobel Biocare) [43,44].

Francois Duret was the first researcher to create the first CAD/CAM restoration in the early 1970s and is recognized as a pioneering figure in the application of CAD/CAM technology in dentistry. The foundation for further advances in dentistry did not begin until 1971, after Duret’s initial descriptions of CAD/CAM systems designed specifically for dental use were documented. This early system was designed to improve the accuracy and efficiency of dental restorations, addressing the limitations of traditional methods that relied heavily on manual craftsmanship. Two years later, Duret presented his approach to dental restorations using CAD/CAM technology at the International Congress of the French Dental Association [10,45].

Bruce Altschuler and Werner Mormann were other contemporaries who contributed to the evolution of CAD/CAM dental technology alongside Duret, thus underlining the importance of Duret’s scientific contributions [2]. Together, these pioneers sought to integrate computer technology into dental practices, representing a revolutionary change from the conventional techniques of the time. Their work contributed significantly to the development of systems for automating the design and manufacturing processes of dental prostheses, thereby improving the quality and consistency of dental restorations [46]. Duret’s innovations have therefore introduced a technological paradigm in dentistry, as CAD/CAM systems have been widely adopted in dental practices worldwide. Since then, the concepts introduced by Duret have been extended, with the technology evolving significantly over the years, improving the ability to design and fabricate dental restorations with high precision and efficiency, resulting in modern CAD/CAM systems that are an integral part of contemporary dental practices, enabling the production of high-quality restorations [43,47]. Therefore, Francois Duret’s early research and the development of CAD/CAM technology in the 1970s were essential for dental practices. His research work not only initiated a new era in dental restorative techniques but also inspired continued advances in the field, leading to the sophisticated systems used in dentistry today.

The capacity and composition of the materials used for those early systems were limited and were often based on metal and ceramic blocks that required extensive machining in the laboratory. However, advances in digital technologies and materials science have led to a wider range of options for chairside applications, enabling immediate restorations during patient visits [48,49]. Later on, the introduction of high-strength ceramics and resin-based materials was essential in the development of in-office CAD/CAM systems. These materials, such as lithium disilicate and zirconia, offer superior mechanical properties and aesthetic qualities compared to traditional materials such as feldspathic porcelain [16,50]. Lee and co-workers showed that CAD/CAM monolithic zirconia exhibits better wear properties than conventional veneering porcelain, which positions it as the preferred material for many dental restorations [51]. Moreover, the flexibility of CAD/CAM technology allows for both permanent and temporary restorations to be fabricated, increasing the efficiency of dental practices [52].

Educational initiatives in dental schools have influenced the clinical integration of chairside CAD/CAM systems, as curricula increasingly include the training of future dentists in digital dentistry [53,54]. This influence is essential, as it prepares future dentists to use CAD/CAM technology effectively, ensuring they can meet the growing demand for aesthetic and functional restorations. Furthermore, surveys indicate that a significant percentage of dental professionals recognize the positive impact of CAD/CAM on the quality of restorations and patient satisfaction [55,56]. Therefore, the adoption of CAD/CAM technology is constantly growing, and its transformative role is reflected in modern dentistry.

### 1.2. Digital Processes for Chairside Oral Rehabilitation

In recent decades, the evolution of digital processes for chairside oral rehabilitation has significantly transformed dental practices. In the beginning, traditional analog methods were most widely used, relying largely on manual techniques for impressions and dental restorations. However, the advent of CAD/CAM systems has marked a major shift towards digital workflows, enabling more efficient and accurate dental procedures. These systems facilitate the immediate fabrication of restorations directly in the dental office, significantly reducing the time required for patient treatment and improving overall workflow efficiency [48,57,58]. Furthermore, research indicates that integrating digital workflows has led to improved clinical outcomes and patient satisfaction. For example, studies have shown that digital impressions have eliminated many inaccuracies associated with conventional methods, such as problems with preparation margins and material handling [59,60]. In addition, the use of intraoral scanners allows for real-time data acquisition, which can be digitally processed to create highly accurate restorations [61,62]. This transition not only streamlines the process but also improves communication between dental professionals and patients, as digital imaging can provide immediate visual feedback on treatment options [63]. Therefore, the emergence of digital workflows in in-office oral rehabilitation has transformed the field of dentistry, in particular for restorative and prosthetic procedures. This statement is also supported by Donker et al. [64], who have shown that digital workflows facilitate immediate implant placement, demonstrating the effectiveness of digital methodologies compared to traditional techniques. Similarly, Cervino et al. [65] emphasized that digital workflows are versatile and applicable in different dental specialties, beneficial for correcting significant bone defects and improving post-extraction alveolar preservation. Moreover, the systematic review reported by Jokstad [66] confirms the advantages of computer-aided technologies in oral rehabilitation, noting that CAD/CAM systems have revolutionized the fabrication of dental restorations. The integration of CAD/CAM technology enables the creation of esthetic restorations in a single program, which is particularly advantageous for both practitioners and patients [67].

Intraoral scanners (IOSs) have significantly transformed the landscape of restorative dentistry, particularly within the context of CAD/CAM technologies. The beginnings of intraoral scanning technology can be traced back to the mid-1980s, when early systems were introduced to facilitate the impression-taking process in dental practices. Over the decades, advancements in optical technology, such as structured light and laser scanning, have enhanced the precision and efficiency of these devices, allowing for direct optical impressions of dental arches and prepared teeth [68,69]. The integration of IOSs into CAD/CAM workflows has led to a paradigm shift, enabling the seamless transition from digital impressions to the fabrication of dental restorations, thus streamlining the overall treatment process [70,71]. The accuracy of intraoral scanners is important, as it directly influences the quality of the final restorations. Various studies have demonstrated that factors such as ambient light conditions, operator skill, and the intraoral environment can significantly affect scanning accuracy [72,73,74]. For instance, Natsubori and co-workers highlighted that improper scanning distances and patient movements could lead to inaccuracies during the scanning process. Moreover, the choice of scanning parameters and the specific technology employed (e.g., active wavefront sampling vs. confocal methods) also play crucial roles in determining the precision of the scans [75]. As the technology continues to evolve, the focus on enhancing the accuracy and reliability of intraoral scanners remains a critical area of research, with implications for both clinical practice and patient outcomes [76,77]. In terms of regulatory considerations, the use of intraoral scanners in clinical settings is subject to various standards and guidelines to ensure patient safety and treatment efficacy. Regulatory bodies, such as the FDA in the United States and the European Medicines Agency (EMA) in Europe, oversee the approval and monitoring of medical devices, including intraoral scanners. These regulations mandate rigorous testing and validation of the devices to confirm their accuracy, reliability, and safety before they can be marketed. Furthermore, ongoing post-market surveillance is essential to monitor the performance of these devices in real-world clinical settings, thereby ensuring that they meet the necessary standards for patient care [71,78]. As the adoption of IOSs continues to grow, adherence to these regulatory frameworks will be important in maintaining high standards of dental practice and patient safety.

Various studies have shown that CAD/CAM technology can be successfully applied in dental practice. For example, Durán et al. and Rinke et al. [79,80] described a step-by-step approach for manufacturing laminate veneers using CAD/CAM technology, emphasizing the reduction in fabrication time and the ability to achieve high-quality results in the dental office. Similarly, Gupta et al. and Durán et al. [79,81] highlighted the successful implementation of CAD/CAM technology in pediatric dentistry, where single-visit restorations have become standard practice, increasing patient comfort and cooperation. In addition, advances in intraoral scanning technologies have significantly improved the accuracy and efficiency of dental procedures. In several research studies, it has been shown that intraoral scanners facilitate the impression process, which is essential for creating accurate 3D models for restorative work [81,82,83]. These technologies not only streamline workflow but also increase patient comfort by minimizing the time spent in the dental office [83].

**Figure 1:**
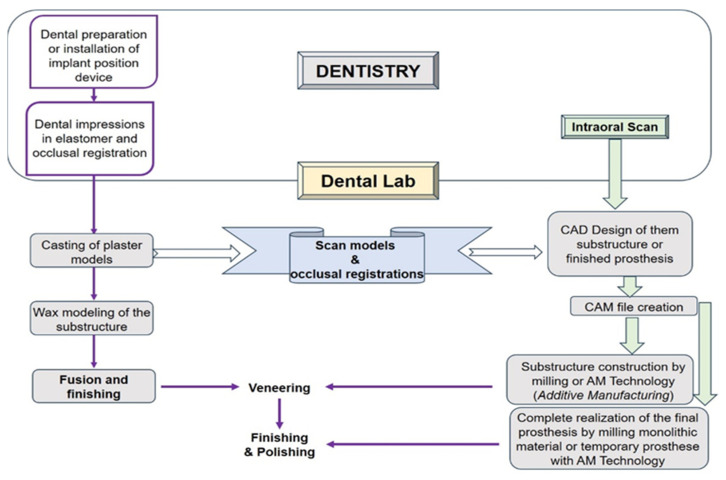
The main manufacturing processes for creating dental restoration.

Recent advances in architectural data generated by CAD software and the subsequent transformation into milling strips for CAM processing have significantly improved the accuracy and efficiency of dental manufacturing processes. The CAD/CAM workflow typically involves three steps: data acquisition, design, and manufacturing (Figure 1). Initially, intraoral scanners or optical scanning techniques capture the geometric data of the dental structures, after which these data are further processed through CAD software to create a virtual pattern. This pattern is then passed to CAM systems to initiate the milling processes, a system that controls the machine in the process of making dentures from solid blocks of material such as zirconia or lithium disilicate [84,85]. The accuracy of this process is essential, as it directly influences the fit and functionality of the final product. Research studies have shown that the accuracy of CAD/CAM systems can be comparable to traditional methods; moreover, in terms of accuracy, some reports have indicated that digital workflows have outperformed conventional techniques [86,87].

Another extremely important element is the choice of milling materials. CAD/CAM systems utilize high-quality materials that exhibit improved mechanical properties such as flexural strength and impact strength compared to conventional materials [88,89]. For instance, Al-Dwairi et al. demonstrated that polymethyl methacrylate (PMMA) processed by CAD/CAM technology exhibits improved performance due to the unique processing conditions used during fabrication [88]. In addition, the milling process itself can be optimized by selecting the appropriate milling tools and parameters, which significantly affect the surface roughness and overall quality of the dentures produced [90].

The integration of advanced technologies, such as photogrammetry and artificial intelligence, into the CAD/CAM workflow, has led to improved accuracy and efficiency in the manufacturing process. Sinada and Papaspyridakos have highlighted the potential of digitally designed verification templates generated from photogrammetric data, emphasizing the importance of accurate impressions for successful results in CAD/CAM applications [86]. Moreover, Tzotzis and co-workers have demonstrated that the development of automated cutting tool design systems improves the milling process by facilitating stress analysis and optimization of tool paths [91].

In the production of dental restorations, the efficiency of the milling axes in CAD/CAM machining devices is an important factor. The number of milling axes directly influences the accuracy, speed, and versatility of the milling process, and these characteristics are essential for the fabrication of high-quality dental restorations; for instance: (i) Three-axis milling is the most common configuration, suitable for simple shapes and simple machining tasks. It allows for cutting in three dimensions but can be limited in terms of producing complicated patterns. (ii) A four-axis milling configuration adds a rotary axis, allowing for the machine to perform more complex cuts and work on multiple parts of a sample without the need for the operator to manually reposition it. (iii) Five-axis milling is an advanced configuration that allows for simultaneous movement along five axes, providing unmatched flexibility and accuracy. It is particularly beneficial for creating complex geometries and intricate patterns that would be difficult or impossible with fewer axes. Research studies indicate that using multi-axis devices, such as five-axis milling machines, can achieve higher accuracy and faster machining times compared to their four-axis counterparts, but with increased cost and complexity regarding the operation process [48,92].

Five-axis milling machines are particularly advantageous because they allow for more complicated geometries and better adaptation of the restoration surface. This capability of milling machines is crucial when fabricating complex frameworks, such as implant-supported prostheses, for which precise fit and adaptation are paramount [48,93]. Compared to five-axis milling machines, the four-axis, although still efficient, may face some problems, leading to possible inaccuracies in the final product [48,92]. At the same time, the choice of milling axis affects the types of materials that can be efficiently machined; for example, larger structures for removable partial dentures are often limited by the capabilities of the milling device [94,95].

**Figure 2:**
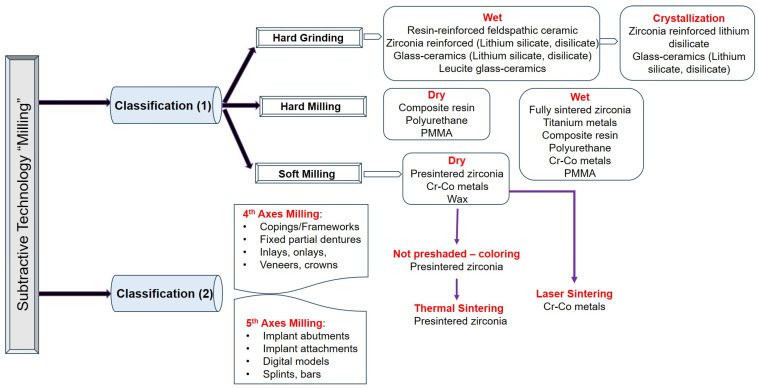
The classification of milling technology according to the type of material used as well as to the number of milling machine axes.

The efficiency of the milling process is determined not only by the number of axes of the milling machine but also by their specific design and the type of materials used (Figure 2). Several studies have shown that different milling tools can have a significant impact on both the surface roughness and the overall quality of the dental restorations produced [90,96]. The integration of advanced CAD software with milling technology further enhances workflow efficiency, allowing for both better design optimization and error reduction during the milling process [97].

Therefore, the number of milling axes in CAD/CAM devices plays a key role in determining the efficiency and quality of dental restorations. Although five-axis systems offer increased capabilities for complex models and improved accuracy, they have higher costs and operational requirements. Therefore, the choice of milling technology must take into account these factors concerning both the specific needs of the dental practice and the types of restorations produced.

### 1.3. Advantages and Disadvantages of In-Office CAD/CAM Technologies

Several advantages of using CAD/CAM technologies in dental practice are highlighted in the literature, particularly in terms of increasing efficiency, improving patient outcomes, and facilitating innovative practices. One of the advantages of CAD/CAM technology is its ability to streamline the restorative process, significantly reducing the time needed for dental procedures. A randomized controlled trial indicated that the time spent applying conventional workflows (148 min) was significantly higher compared to the time required to apply CAD/CAM processes (74–92 min) [98]. This efficiency not only increases patient satisfaction but also optimizes workflow in dental practices [99].

Another advantage of CAD/CAM technology is the accuracy and precision of dental restorations. The digital workflows involved in CAD/CAM technology enable the meticulous design and fabrication of dentures, which improves the fit and esthetic quality of restorations. Mounajjed and co-workers have demonstrated that restorations created using CAD/CAM systems exhibit superior marginal fit compared to those produced using traditional methods [100]. In addition, CAD/CAM technologies enable the production of high-strength ceramics that meet the growing demand for esthetic and durable restorations essential in modern dentistry [47].

Another advantage of this technology is the integration of CAD/CAM systems into contemporary challenges (such as the COVID-19 pandemic, for example), which has led to the development of innovative solutions by manufacturing customized protective equipment, thus enhancing biosafety in dental practices [61]. This adaptability emphasizes the role of CAD/CAM technologies not only in routine dental procedures but also in addressing emerging healthcare needs. In addition, the increasing acceptance and use of CAD/CAM technologies by dental professionals indicates a shift toward digital dentistry. Statistics report that a significant percentage of dentists are incorporating CAD/CAM systems in their practices, while another percentage express interest in further integration [55,56]. This trend reflects a growing recognition of the advantages offered by CAD/CAM technology, including improved patient outcomes, reduced clinical time, and lower laboratory costs [43,101].

Although CAD/CAM technologies have revolutionized dental practice by enabling the rapid production of dental restorations, their implementation is not without drawbacks, which can affect both clinical outcomes and operational efficiency. In the following, some disadvantages of using these revolutionary technologies are presented.

One of the significant disadvantages of CAD/CAM systems in dental practice is the high cost and current expenses. Purchasing CAD/CAM equipment requires a substantial financial investment, which can be a barrier for many dental practices, especially small ones. The costs associated with CAD/CAM technology are higher than for traditional methods, mainly due to the expense of the equipment and materials used. In addition, operational costs may increase due to the need for specialized materials and maintenance of the technology [102].

Another disadvantage is the implementation of the learning process for using CAD/CAM systems. Dental professionals often need extensive training to use these technologies effectively, which can take several months. This learning process can lead to inefficiencies and errors in the production of restorations, which can compromise the quality of patient care during the transition period. In addition, the complexity of software and hardware may discourage many practitioners from fully integrating CAD/CAM technology into their workflows, which can lead to inconsistent use and underutilization of the technology’s capabilities [103].

Quality control is also a disadvantage for CAD/CAM systems in dental practices. Although technological advances have improved the accuracy of restorations, problems such as poor marginal fit and internal adaptation of the restoration can still occur, especially if the scanning and milling processes are not executed correctly [104]. This can lead to a higher rate of re-dos or adjustments, which not only affects patient satisfaction but also increases the time and overall cost of treatment. In addition, the reliance on digital workflows may lead to decreased utilization of traditional workflows among dental professionals, which could negatively influence their overall competence in restorative dentistry [105].

The use of non-conforming materials in CAD/CAM systems can present another disadvantage. For example, some CAD/CAM materials may be susceptible to degradation following treatments in dental practices, such as bleaching agents, which can affect their hardness and surface properties [106]. This susceptibility raises questions about the long-term durability and aesthetic outcomes of restorations produced using CAD/CAM technology.

Therefore, the benefits of CAD/CAM technologies in dental practices are multiple, including increased efficiency and accuracy, as well as the ability to respond to contemporary challenges in dental practice. As the adoption of these technologies continues to grow, the likelihood that they will play a determining role in shaping the future of dental care is very high. Although CAD/CAM technologies offer many advantages from their use in dental practices, they are also accompanied by significant disadvantages, including high costs, a rapid learning process, potential quality control issues, and the limitations of certain materials. These factors should be carefully considered by dentists when deciding to implement CAD/CAM systems in their practices.

## 2. In-Office CAD/CAM Materials

**Figure 3:**
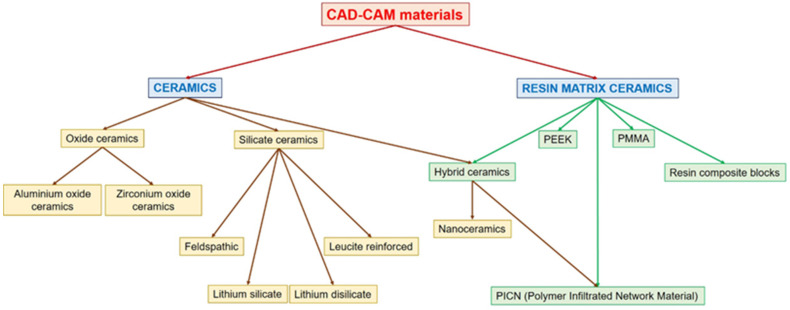
Classification of CAD/CAM materials.

The advent of chairside CAD/CAM technology has revolutionized the field of restorative dentistry, allowing for the efficient and precise fabrication of dental restorations directly within the clinical setting. The materials used in chairside CAD/CAM systems can be broadly categorized into ceramics, hybrid ceramics, and polymer-based materials, each exhibiting unique characteristics that influence their application in dental restorations. Figure 3 presents a detailed classification of CAD/CAM materials used in dentistry.

Ceramic materials, particularly lithium disilicate and zirconia, are among the most widely used in chairside CAD/CAM systems due to their superior mechanical properties and aesthetic qualities. Lithium disilicate, known for its excellent transparency and strength, has been shown to perform well in clinical settings, with studies indicating low rates of esthetic complications and high fracture resistance [107,108]. Zirconia, particularly in its fully sintered form, has also demonstrated commendable mechanical properties, making it suitable for single-visit dentistry [109,110]. The mechanical performance of these materials is critical, as they must withstand the functional loads encountered in the oral environment. Research has confirmed that CAD/CAM ceramics exhibit fracture resistance comparable to traditional materials, with fracture loads reaching up to 1700 N for certain CAD/CAM crowns [111].

Hybrid ceramics and polymer-based materials represent another significant category within chairside CAD/CAM systems. Hybrid ceramics, which combine the benefits of both ceramics and polymers, have been shown to possess favorable mechanical properties, including enhanced toughness and reduced brittleness [106,112]. These materials are particularly advantageous in situations where esthetics and strength are both critical, such as in anterior restorations. Polymer-based materials, particularly those reinforced with fillers such as graphene, have emerged as promising alternatives due to their lightweight nature and ease of manipulation [112]. The mechanical properties of these materials, including flexural strength and impact resistance, have been shown to be superior to conventional heat-cured polymers, making them suitable for various restorative applications [88].

The microstructural characteristics of CAD/CAM materials play a significant role in determining their performance. Scanning Impulse Acoustic Microscopy (SIAM) has been employed to investigate the internal structure of various chairside CAD/CAM materials, revealing significant differences in microstructure among ceramic, hybrid ceramic, and polymeric materials [113]. These microstructural variations can influence not only the mechanical properties but also the aesthetic outcomes of restorations. For instance, the surface roughness of CAD/CAM materials has been shown to affect microbial adherence, with smoother surfaces demonstrating lower levels of plaque accumulation and gingival inflammation [88,114]. This is particularly relevant in the context of long-term clinical performance, as the surface properties of materials can significantly impact their longevity and the health of surrounding tissues.

In terms of clinical application, the choice of CAD/CAM material is often influenced by the specific requirements of the restoration being fabricated. For example, the study by Hu et al. revealed that in cases involving endodontically treated teeth, the mechanical properties of the chosen material are critical, as these restorations must endure significant occlusal forces [115]. Studies have indicated that chairside CAD/CAM restorations can be a cost-effective and time-efficient alternative to traditional methods, allowing for the fabrication of high-quality restorations in a single visit [22,48]. This efficiency not only enhances patient satisfaction but also streamlines clinical workflows, making CAD/CAM technology an attractive option for modern dental practices.

The integration of CAD/CAM technology into dental education has also been noteworthy, with many institutions adapting their curricula to include training on these advanced systems. This shift is important, as proficiency in utilizing CAD/CAM technology is becoming increasingly essential for dental professionals. The ability to effectively employ CAD/CAM systems can significantly impact the quality of care provided to patients, as well as the overall efficiency of dental practices [53].

Despite the numerous advantages of chairside CAD/CAM materials, challenges remain in terms of their long-term performance and clinical outcomes. Factors such as aging, staining, and the effects of various cementation strategies can influence the durability and aesthetic properties of CAD/CAM restorations [116,117]. Ongoing research is essential to address these challenges and to further refine the materials and techniques used in CAD/CAM dentistry.

Commercially available CAD/CAM materials have significantly advanced in recent years, enhancing the quality and efficiency of dental restorations. Notable examples include lithium disilicate ceramics, such as IPS e.max by Ivoclar Vivadent, which are renowned for their excellent esthetics and mechanical properties [118]. Another prominent material is monolithic zirconia, exemplified by brands like BruxZir, which offers superior strength and reduced risk of veneer chipping, making it ideal for posterior restorations [51]. Additionally, hybrid ceramics, such as Cerasmart 270, combine the benefits of ceramics and polymers, providing high micromechanical properties suitable for various applications [112]. These materials reflect the ongoing evolution in CAD/CAM technology, which continues to improving the precision and aesthetic outcomes of dental restorations [43,119]. Table 1 presents a comprehensive overview of commercially available CAD/CAM materials.

### 2.1. Feldspathic Ceramics and Leucite-Reinforced Ceramic Blocks

In the realm of in-office CAD/CAM materials, feldspathic ceramics and leucite-reinforced ceramics hold significant importance due to their aesthetic qualities. These materials are particularly favored for their ability to mimic the natural appearance of teeth, making them ideal for anterior restorations. Indications for their use include anterior and posterior crowns, inlays, and veneers, where aesthetics and translucency are crucial [120,121,122]. However, contraindications may arise in cases requiring high-strength restorations, as feldspathic ceramics generally exhibit lower flexural strength compared to reinforced options. The mechanical properties of these ceramics are influenced by their composition and processing. Studies indicate that while both types exhibit similar resilience moduli, the machining process can introduce residual stresses that may lead to failure [123,124]. Surface treatments, such as hydrofluoric acid etching, are critical for enhancing bonding strength with resin cements, although optimal protocols remain debated [125,126].

Feldspathic ceramics, characterized by their glassy matrix and crystalline structure, have been utilized in dentistry for over a century. They consist primarily of silica, alumina, and potassium oxide, which contribute to their translucency and aesthetic appeal [127,128]. The aesthetic properties of feldspathic ceramics are attributed to their high glass content, which allows for light transmission and color matching to that of natural tooth structure, thus providing a lifelike appearance in restorations [127,129].

Leucite-reinforced ceramics, on the other hand, are a subclass of feldspathic ceramics that incorporate leucite crystals into their matrix. This addition enhances their mechanical strength while maintaining their aesthetic qualities. The presence of leucite increases the flexural strength of these materials, making them more suitable for applications requiring greater durability, such as posterior restorations [120,130]. Studies have shown that leucite-reinforced ceramics exhibit improved fracture resistance compared to traditional feldspathic ceramics, which is critical in clinical settings where restorations are subjected to significant occlusal forces [131,132]. The mechanical properties of leucite-reinforced ceramics, including flexural strength and fracture toughness, have been reported to be superior to those of conventional feldspathic ceramics, making them a preferred choice for many dental practitioners [120,130].

The processing of these ceramics through CAD/CAM technology has further enhanced their clinical applicability. The ability to mill these materials chairside allows for the fabrication of restorations in a single visit, significantly improving patient convenience and satisfaction [133]. Recent advancements in CAD/CAM technology have also led to the development of improved milling techniques that enhance the surface quality and mechanical properties of these materials. The milling process for feldspathic and leucite-reinforced ceramics is designed to achieve precise fit and finish, which is essential for the longevity and performance of dental restorations [128,129].

In terms of bonding and cementation, both feldspathic and leucite-reinforced ceramics require specific surface treatments to optimize their adhesion to resin cements. Hydrofluoric acid etching is commonly employed to enhance the surface roughness, thereby increasing the bonding strength between the ceramic and the resin. The bond strength of these materials can be influenced by various factors, including the etching time and the type of resin cement used [125]. The research by Ellakany et al. indicates that proper surface treatment can significantly improve the longevity of restorations made from these ceramics, particularly in challenging clinical scenarios such as those involving discolored teeth [134].

Moreover, the translucency of feldspathic and leucite-reinforced ceramics is a critical factor in achieving aesthetic outcomes. The high glass content in feldspathic ceramics contributes to their translucency [127,129]. Leucite-reinforced ceramics, while also exhibiting good translucency, tend to have slightly lower translucency compared to feldspathic ceramics due to the crystalline structure introduced by leucite [135,136]. This difference can influence the choice of material based on the specific aesthetic requirements of the restoration.

The clinical performance of feldspathic and leucite-reinforced ceramics has been extensively studied. Studies indicate favorable outcomes in terms of fracture resistance and wear characteristics. For instance, feldspathic ceramics have demonstrated good wear resistance against opposing dental materials, although they can be abrasive if not polished adequately, as reported by Yanishen et al. and Gwon et al. [128,137]. Leucite-reinforced ceramics, due to their enhanced mechanical properties, show improved resistance to fracture and wear, making them suitable for a wider range of applications, including posterior restorations where occlusal forces are greater [120,132,138].

Feldspathic ceramics and leucite-reinforced ceramics are integral components of in-office CAD/CAM materials, offering a balance of aesthetic appeal and mechanical strength. Their unique properties, combined with advancements in CAD/CAM technology, enable dental practitioners to deliver high-quality restorations efficiently.

### 2.2. Lithium Disilicates and Zirconia-Reinforced Lithium Silicate Blocks

Lithium disilicate and zirconia-reinforced lithium silicate are important materials in contemporary CAD/CAM technology. Their unique properties make them suitable for various restorative applications, including crowns, veneers, and bridges.

Lithium disilicate is a glass–ceramic material that has gained popularity due to its excellent aesthetic properties and mechanical strength. It is primarily composed of lithium silicate crystals embedded in a glassy matrix, which contributes to its translucency and color stability [20]. The material exhibits a flexural strength ranging from 360 to 400 MPa, making it suitable for anterior and posterior restorations [139]. The microstructure of lithium disilicate consists of needle-like crystals that enhance its mechanical properties, particularly its fracture toughness, allowing for the material to withstand occlusal forces without significant risk of fracture [140].

Zirconia-reinforced lithium silicate, on the other hand, combines the aesthetic advantages of lithium disilicate with the superior mechanical properties of zirconia. The addition of zirconia enhances the strength of the material, with reported values exceeding 1000 MPa [139]. This makes zirconia-reinforced lithium silicate particularly advantageous for posterior restorations where higher strength is required. The incorporation of zirconia also improves the material’s resistance to chipping and cracking, which is a common failure mode in all-ceramic restorations [141]. Furthermore, the study by Velastegui et al. reveals that the presence of zirconia contributes to a more favorable wear behavior against opposing dentition, thus preserving the integrity of the antagonist’s teeth [142].

The optical properties of both materials are critical for achieving aesthetic outcomes in restorative dentistry. Studies have shown that lithium disilicate exhibits a higher translucency compared to zirconia-reinforced lithium silicate, which may be beneficial in anterior restorations where esthetics are paramount [143]. However, zirconia-reinforced lithium silicate has been reported to have improved color stability under various conditions, making it a reliable choice for long-term restorations [144]. The translucency and color stability of these materials can be influenced by factors such as surface finishing and the thickness of the restoration [20,21].

In terms of machinability, lithium disilicate demonstrates superior milling characteristics compared to zirconia-reinforced lithium silicate, which is essential for efficient chairside CAD/CAM applications. The milling accuracy of lithium disilicate is reported to be higher, allowing for a precise fit and adaptation of restorations [87]. However, the mechanical properties of zirconia-reinforced lithium silicate may compensate for its lower machinability, as the material’s strength allows for thinner restorations without compromising durability [139].

Biocompatibility is another critical aspect of dental materials. Both lithium disilicate and zirconia-reinforced lithium silicate have been shown to exhibit favorable biocompatibility profiles, with cell viability above 90% indicating non-cytotoxic behavior [145]. This biocompatibility and the chemical composition of these materials is particularly important in restorative dentistry, where materials are in direct contact with oral tissues, the absence of harmful substances contributing to their biosafety and acceptance in clinical practice [146].

The clinical implications of using lithium disilicate and zirconia-reinforced lithium silicate are significant due to their mechanical and aesthetic properties, making them suitable for a wide range of applications, from single-unit restorations to complex multi-unit prostheses. The choice between the two materials often depends on the specific clinical scenario, including the location of the restoration, the patient’s occlusal forces, and aesthetic demands. For instance, lithium disilicate is often preferred for anterior restorations due to its superior translucency, while zirconia-reinforced lithium silicate may be chosen for posterior applications where strength is more important [139,147].

A widely used procedure in prosthetic dentistry is represented by ceramic laminate veneers, which provide attractive aesthetics. The most commonly used materials are feldspathic porcelain; leucite-reinforced glass ceramics (LRGC); lithia-based glass ceramics, mainly lithium disilicate (LDS); and resin matrix ceramics (RMC). The ability of LRGC and feldspathic porcelain to replicate a genuine tooth with its own tint and translucency makes them aesthetically superior. Both materials, however, are linked to poor mechanical qualities [148]. A systematic review conducted by Klein and Spitznagel concluded that using leucite-reinforced glass ceramic, lithium disilicate ceramic, and feldspathic ceramic to create ceramic laminate veneers is a dependable and minimally invasive treatment option with good long-term outcomes. Regarding technical and biological problems, lithium disilicate ceramic performs marginally better than feldspathic and leucite-reinforced glass ceramic laminate veneers. Zirconia laminate veneers’ long-term performance is still unknown [149].

Moreover, it is important to consider the oral environment when selecting materials for restorative procedures, with studies revealing that both materials exhibit changes in fracture resistance over time, particularly when exposed to acidic environments [139].

### 2.3. Zirconium Oxide

Zirconium oxide, commonly referred to as zirconia, has emerged as an important material in dentistry, particularly in the realm of prosthodontics and implantology. Its unique properties, including high mechanical strength, fracture resistance, biocompatibility, and aesthetic appeal, have made it a preferred choice for various dental applications, including crowns and fixed prostheses. The material’s aesthetic qualities are also noteworthy; zirconia can achieve a natural tooth-like appearance, which is essential for restorations in visible areas. Moreover, CAD-CAM technology enhances the efficiency of zirconia fabrication, allowing for precise restorations while reducing costs and material waste. The ability to produce monolithic zirconia restorations eliminates the need for veneering, thus minimizing common complications such as veneer chipping. Additionally, zirconia’s chemical stability and corrosion resistance further contribute to its longevity and reliability in dental applications [150,151,152,153].

Zirconium oxide is characterized by its excellent mechanical properties, which include high flexural strength and fracture toughness. These attributes are particularly beneficial in dental applications where durability is essential. Studies have shown that zirconia ceramics, especially those stabilized with yttria polycrystals (3Y-TZP), exhibit remarkable mechanical performance, making them suitable for load-bearing restorations such as crowns and bridges [154,155]. The study by Al-Saleh et al. reported that the mechanical properties of zirconia can be further enhanced through various processing techniques, including sintering and the incorporation of nanoparticles, which can improve the material’s overall strength and resilience [156].

Zirconium oxide exhibits properties that closely resemble natural tooth enamel, allowing for superior aesthetic outcomes in restorative dentistry. However, it is important to note that while zirconium oxide provides excellent strength, its translucency can be limited compared to other ceramic materials. As such, it is often recommended to use zirconia in conjunction with veneering ceramics to achieve optimal esthetic results. According to the study by Špehar and Jakovac, this combination allows for clinicians to effectively exploit the mechanical strength of zirconia while enhancing the visual appeal of the restoration [157].

The versatility of zirconium oxide extends to its various forms and applications in dentistry. It is commonly used in the fabrication of crowns, bridges, implant abutments, and even orthodontic brackets [155,158]. The advent of CAD/CAM technologies has revolutionized the way zirconia restorations are produced, allowing for precise customization and efficient fabrication processes. This digital workflow not only streamlines the production of dental restorations but also enhances the accuracy of fit and finish, ultimately improving patient outcomes [159].

Despite its numerous advantages, zirconium oxide has its limitations. One of the primary challenges associated with zirconia is its brittleness, which can lead to catastrophic failure under certain conditions. Research has indicated that while zirconia exhibits high strength, it is susceptible to crack propagation, particularly in thin sections [157]. To mitigate this risk, future studies should explore various reinforcement strategies, such as the incorporation of other materials and the optimization of processing conditions to enhance the toughness of zirconia ceramics.

While many studies report favorable outcomes, there is still a need for long-term performance studies in clinical settings for a full understanding of the durability and longevity of zirconia-based restorations [154,160]. The integration of zirconia in dental practice also needs careful consideration of its bonding properties, as achieving a reliable bond between zirconia and resin cements can be challenging [161,162]. Surface treatments and modifications to enhance adhesion and improve the overall performance of zirconia restorations are recommended by Al-Saleh and co-workers [156].

### 2.4. The Hybrid Ceramic (Resin Matrix Ceramics) Materials

Hybrid ceramics, also known as resin matrix ceramics, represent a significant advancement in dental materials, particularly in the context of chairside CAD/CAM systems. These materials combine the desirable properties of ceramics and polymers, offering improved mechanical performance, aesthetic qualities, and ease of use in clinical settings. The classification of these materials typically includes polymer-infiltrated ceramic networks (PICNs), resin nanoceramics, and hybrid ceramics, each with unique characteristics and applications in restorative dentistry [125,144,163].

The fundamental structure of hybrid ceramics consists of a ceramic framework that is infiltrated with a resin matrix. This dual-phase composition allows for enhanced mechanical properties, such as increased flexural strength and reduced brittleness, compared to traditional ceramics. For instance, Vita Enamic, a well-known hybrid ceramic, features a porous ceramic network filled with a polymer, creating a double-network structure that contributes to its resilience under functional loads [164,165]. This innovative design not only improves the material’s toughness but also mimics the mechanical behavior of natural tooth structures, making it a suitable choice for various restorative applications [165].

Concerning aesthetic properties, hybrid ceramics exhibit favorable optical characteristics, including translucency and color stability. Several studies have shown that the color stability of hybrid ceramics can be superior to that of traditional ceramics, particularly when subjected to staining agents. For example, research conducted by Seyidaliyeva et al. and Amri et al. indicates that polymer-infiltrated ceramics demonstrate less discoloration compared to conventional ceramics, which is critical for maintaining the aesthetic integrity of dental restorations over time [144,166]. However, it is essential to note that the resin content within these materials can influence their color stability. Aydın and co-workers noted that higher resin content may lead to increased susceptibility to color changes [167].

The surface properties, such as surface roughness, of hybrid ceramics also play an important role in their clinical performance because they can affect plaque accumulation and aesthetic appearance. Studies have shown that different finishing and polishing techniques can significantly impact the surface roughness of hybrid ceramics, influencing their long-term performance and aesthetic outcomes [167,168]. Furthermore, the mechanical wear resistance of these materials is comparable to that of traditional ceramics, making them suitable for high-stress applications in the oral environment [165].

Another critical aspect of hybrid ceramics includes their bonding characteristics. The adhesion of resin cements to these materials is influenced by surface treatments, such as etching with hydrofluoric acid, which enhances micromechanical retention. The bond strength between hybrid ceramics and resin composites is essential for the longevity of restorations, and various studies have explored the optimal bonding protocols to maximize this strength [169,170]. The introduction of universal adhesives has further simplified the bonding process, allowing for more versatile clinical applications [171].

Hybrid ceramics also demonstrate favorable biocompatibility, which is essential for materials used in direct contact with oral tissues. The interaction of these materials with fibroblast cells has been studied to assess their biological response, indicating that they are well tolerated in the oral environment. This biocompatibility, combined with their mechanical and aesthetic properties, positions hybrid ceramics as a promising option for a wide range of restorative procedures, including crowns, inlays, and veneers [146].

The versatility of hybrid ceramics extends to their use in various clinical scenarios, from single-unit restorations to complex multi-unit cases. Their ability to be milled chairside allows for efficient treatment workflows, reducing the need for multiple appointments and enhancing patient satisfaction [164,165]. Moreover, CAD/CAM technology advancements have facilitated the precise fabrication of these materials, ensuring a high level of fit and finish in restorations [172].

### 2.5. Acrylic Resin

Acrylic resin, especially polymethyl methacrylate (PMMA), is a fundamental material in dentistry, particularly for creating removable prostheses like dentures and orthodontic devices. The versatility of PMMA is underscored by its favorable mechanical properties, aesthetic qualities, and biocompatibility, which have made it the material of choice since its introduction in the 1930s [173]. The chemical structure of PMMA allows for a variety of modifications, enhancing its performance in clinical applications [174].

The classification of acrylic resins is primarily based on their chemical structure, which can be divided into methacrylate acrylic resins and bis-acryl composite resins. Among these, PMMA is the most widely used due to its excellent mechanical and aesthetic properties [175]. The material’s high molecular weight contributes to its strength and durability, making it suitable for applications that require resilience under stress, such as denture bases. Furthermore, the crosslinking of PMMA enhances its resistance to chemical degradation, which is important for maintaining the integrity of dental appliances exposed to various oral conditions [176].

In terms of processing, acrylic resins can be polymerized through heat or autopolymerization methods. Heat-cured acrylic resins are commonly employed for denture bases due to their superior mechanical properties compared to cold-cured variants. However, the porosity associated with heat-cured acrylics can lead to complications such as microbial colonization and staining, which are critical considerations in clinical practice [177]. Recent advancements in additive manufacturing technologies have also introduced new avenues for utilizing acrylic resins in dentistry. These technologies allow for the production of customized dental appliances with high precision and reduced fabrication time, further expanding the applications of acrylic materials [178,179].

The aesthetic properties of acrylic resins are particularly noteworthy, as they can be tinted to match the natural color of the gums and teeth, providing a more natural appearance for dental prostheses [180]. However, factors such as exposure to staining agents (e.g., coffee, tea, nicotine) can adversely affect the color stability of acrylic resins over time [181,182]. Studies have shown that the surface finish and polishability of acrylic resins play a significant role in their susceptibility to staining, with smoother surfaces exhibiting less discoloration [181].

Moreover, the mechanical properties of acrylic resins, such as flexural strength and impact resistance, are crucial for their performance in dental applications. The mechanical behavior of PMMA can be influenced by various factors, including the thickness of the material and the processing methods employed [183]. Bond strength in PMMA can be influenced by several factors, including chemical composition and type of resin, polymerization type and temperature used, manufacturing method, surface treatment, presence of additives, temperature and time, disinfectants and cleaning agents, mechanical interlocking, or allergic reactions [184,185]. For instance, the study by Clark and Hsu has demonstrated that the bond strength between denture teeth and the acrylic base can be affected by the thickness of the acrylic layer, highlighting the importance of proper material selection and processing in achieving optimal clinical outcomes [186].

In addition to traditional applications, acrylic resins are increasingly being explored for innovative uses in dentistry. For example, their role in the fabrication of interim prosthetic restorations using 3D printing technologies has gained attention due to their ability to create highly customized and precise dental appliances [187,188]. The mechanical properties of 3D-printed acrylic resins are comparable to those of conventionally processed materials, making them a viable alternative in clinical settings [183].

Furthermore, the development of flexible acrylic resins has opened new possibilities for enhancing the comfort and retention of removable dental appliances. Flexible acrylics can adapt to the contours of the oral cavity, providing improved fit and patient satisfaction. This adaptability is particularly beneficial in cases where traditional rigid materials may not provide adequate retention due to anatomical variations [189].

## 3. Optical Properties and Long-Term Aesthetic Durability of the CAD/CAM Materials

In the realm of restorative dentistry, the selection of appropriate chairside CAD/CAM materials is very important for achieving optimal functional and aesthetic outcomes. Natural glance and individualization hold a unique place in today’s world, where doctors and patients seek out exceptional aesthetic outcomes due to the promotion of mass media and the Internet. This aspiration remains one of the most difficult problems with CAD/CAM materials: achieving a natural tooth look while maintaining adequate mechanical strength. A study that evaluated the optical properties of contemporary monolithic CAD/CAM materials with different chemical compositions concluded that optical properties are in direct correlation with the type and thickness of the material used, as well as clinical relevance monolithic materials should be carefully selected to provide aesthetic restorations and shade matching with the natural dentition, particularly for anterior teeth [190].

Over time, certain questions have been expressed about the use of CAD/ CAM blocks. One of them regarding their monochromatic nature. The materials are also available on polychromatic blocks (Vitablocs Triluxe forte and Vitablocs RealLife, Vita ZahnFabrik). Vitablocs TriLuxe (Vita Zahnfabrik), which has a graded variation in color saturation (three shades), is an option for a clinician who is still worried about the monochromatic character of these blocks that allows for the replication of a natural tooth’s optical properties, including color intensity and translucency, which could improve the restoration’s integration with the remainder of the natural dentition [122].

Lithium disilicate ceramics, such as IPS e.max, are renowned for their superior aesthetic qualities, which closely mimic natural tooth structure. These materials exhibit excellent translucency and color stability, making them suitable for anterior restorations, including veneers and crowns [5]. Studies have shown that lithium disilicate restorations maintain their aesthetic appeal over time, with minimal color change and high resistance to staining [144]. Furthermore, the optical properties of lithium disilicate allow for effective light transmission, which is essential for achieving lifelike aesthetics in dental restorations [191]. The long-term performance of lithium disilicate has been well documented, with high survival rates reported in clinical studies, thus reinforcing its status as a preferred material for aesthetic restorations [11].

Zirconia-reinforced ceramics, on the other hand, offer a different set of advantages. These materials are characterized by their high strength and durability, making them ideal for posterior restorations where functional demands are greater [192]. The challenge lies in balancing strength and aesthetics, as zirconia’s opacity can sometimes lead to less natural-looking restorations, particularly in anterior applications [193]. Nevertheless, zirconia’s excellent mechanical properties and resistance to fracture make it a valuable option for specific clinical situations, especially where strength is necessary [194]. The results of the investigation conducted by Sen and co-workers showed that zirconia-reinforced glass ceramics performed better in terms of biaxial flexural strength than dual-network ceramic, feldspathic ceramic, lithium disilicate ceramic, and resin nanoceramic [195]. The conclusion of another study was that generally speaking, zirconia-based ceramic materials have better compressive strength qualities and better optical qualities than lithium disilicate-based ceramics [196].

Hybrid ceramics, which combine the properties of ceramics and polymers, represent a newer category of CAD/CAM materials. These materials, such as Cerasmart and Enamic, exhibit a unique combination of aesthetics and mechanical properties, making them versatile for various restorative applications [197]. Hybrid ceramics generally demonstrate good color stability and lower susceptibility to staining compared to traditional ceramics, which is a significant advantage in maintaining aesthetic outcomes over time [167]. However, their optical properties can vary significantly based on their resin content and the specific formulation used [198]. For instance, studies have indicated that hybrid ceramics with higher resin content may experience more color change than those with lower resin content, highlighting the importance of material selection based on clinical requirements [199].

A randomized clinical study that evaluated the performance of ceramic, hybrid, and composite CAD/CAM materials indicated that all three types exhibited favorable optical properties and aesthetic durability over the 30-month follow-up period. The restorations maintained their surface brightness, color harmony, and anatomical form, suggesting that they retained their aesthetic qualities effectively during this time. While the study did not specify a single material as having the best results in terms of aesthetic performance, it did report that there were no statistically significant differences in the aesthetic criteria evaluated among the three groups (ceramic, hybrid, and composite) after 30 months. This implies that all materials performed comparably well in terms of aesthetics over the follow-up period [200].

The study by Demirel and co-workers highlights that resin nanoceramic CAD/CAM materials exhibit excellent optical properties, closely mimicking the translucency and color of natural teeth, which is essential for achieving aesthetic restorations. Their long-term aesthetic durability is superior to traditional composite materials, as they resist wear and staining, maintaining their appearance over time. These properties significantly influence clinical decisions, as clinicians are more likely to choose these materials for restorations where aesthetics and longevity are critical. Consequently, the enhanced aesthetic outcomes and durability contribute to higher patient satisfaction, as patients are more pleased with restorations that retain their natural look and function throughout the years [201].

The study conducted by Karaokutan and co-workers investigated three types of CAD/CAM materials used for inlay restorations comprising feldspathic ceramic, resin nanoceramic, and leucite glass–ceramic and highlighted the distinct optical properties and aesthetic durability, which are critical in restorative dentistry. Known for its excellent optical properties, feldspathic ceramics mimic the natural translucency and color of teeth. They have a high aesthetic appeal due to their ability to reflect light similar to natural enamel. However, they may be more susceptible to wear and staining over time compared to other materials. Resin nanoceramic material combines the aesthetic qualities of ceramics with the toughness of resin composites. While it offers good polishability and initial color stability, the study found that it exhibited the highest color change (ΔE = 9.29) after accelerated artificial aging, making it less aesthetically durable in the long term. The optical properties can be affected by the aging process, leading to increased opacity and undesirable color shifts. Leucite glass–ceramic material strikes a balance between aesthetics and durability. It has good optical properties, similar to feldspathic ceramics, and demonstrated moderate color stability (ΔE = 2.46) after accelerated artificial aging. Leucite glass–ceramics are less prone to color changes compared to resin nanoceramics, making them a reliable choice for long-term restorations [202].

Moreover, the distinction between monochromatic and polychromatic CAD/CAM materials is important for clinicians, as it directly influences the aesthetic outcomes of dental restorations. A monochromatic material consists of a single shade throughout its bulk, while a polychromatic material exhibits variations in shade and translucency across its layers or within its structure, better mimicking the natural tooth. These material characteristics play a significant role in how restorations respond to intraoral conditions. Changes in translucency and color stability are key indicators of material degradation, which can affect both the aesthetic appeal and functional longevity of the restoration.

Feldspathic ceramics are typically monochromatic, which can limit their ability to mimic the natural gradient of tooth color. Studies have shown that feldspathic ceramics exhibit high translucency, which is essential for achieving a natural appearance in anterior restorations [140]. However, their color stability can be affected by factors such as surface treatments and aging, leading to potential aesthetic concerns over time [202].

Leucite-reinforced ceramics are also predominantly monochromatic and are known for their good aesthetic properties and mechanical strength. They exhibit higher translucency compared to traditional feldspathic ceramics, making them suitable for anterior restorations [203]. However, their color stability can be compromised under certain conditions, such as exposure to staining agents, which can lead to noticeable color changes [202]. The translucency of leucite-reinforced ceramics is influenced by their microstructure, which can scatter light differently based on the thickness and surface finish [204].

Lithium disilicates are recognized for their high translucency and strength, making them a popular choice for both anterior and posterior restorations. These materials can be produced in both monochromatic and polychromatic forms, with the latter providing a more natural gradient of color that mimics the optical properties of natural teeth [205]. Research indicates that lithium disilicate ceramics maintain their color stability better than other materials when subjected to aging processes, although they can still exhibit some discoloration over time [202]. The translucency of lithium disilicates is generally superior, allowing for effective light transmission, which is critical for aesthetic restorations [206].

Zirconia-reinforced lithium silicate combines the strength of zirconia with the aesthetic properties of lithium silicate. This material is often available in both monochromatic and polychromatic options, enhancing its versatility in clinical applications. Studies have shown that zirconia-reinforced lithium silicate displays excellent color stability and translucency, making it suitable for various restorative procedures [207,208]. The presence of zirconia enhances the mechanical properties, while the lithium silicate component contributes to its aesthetic qualities [208].

Zirconium oxide is typically opaque and primarily used for frameworks in dental restorations. While it is not known for its aesthetic properties, advancements have led to the development of translucent zirconia options that can be used in monolithic restorations. These materials, however, are generally monochromatic and may not provide the same level of aesthetic appeal as other materials like lithium disilicate [209]. The translucency of zirconia can be enhanced through specific processing techniques but it still lacks the natural gradient found in polychromatic materials [210].

Hybrid ceramics, such as Vita Enamic, combine the properties of ceramics and polymers, offering a unique solution for aesthetic restorations. These materials are available in both monochromatic and polychromatic forms, allowing for enhanced aesthetic outcomes. The translucency of hybrid ceramics can be adjusted based on their composition and processing, making them suitable for a variety of clinical scenarios [211,212]. Studies indicate that hybrid ceramics maintain good color stability, although they may be more susceptible to staining compared to traditional ceramics [213].

Acrylic resin is often used in temporary restorations and can be produced in various shades. While acrylic resins can be made to mimic the appearance of natural teeth, they are generally less stable in terms of color and translucency compared to ceramic materials. Acrylics are typically monochromatic, which can limit their aesthetic potential in long-term applications. The color stability of acrylic resins can be affected by environmental factors, leading to discoloration over time [202].

Table 2 presents a comprehensive overview of CAD/CAM materials used in restorative dentistry, focusing on their optical properties, durability, and aesthetic considerations.

## 4. The Properties of In-Office CAD/CAM Materials

The properties of in-office CAD/CAM materials are important factors for their successful application in modern dentistry. These materials, which include various ceramics, composites, and polymers, have been developed to meet the demands for aesthetics, durability, and efficiency in restorative procedures. Understanding their mechanical, physical, and aesthetic properties is essential for optimizing their use in clinical practice.

One of the primary advantages of CAD/CAM materials is their mechanical strength. Research indicates that hybrid ceramic-based CAD/CAM restorations exhibit superior micromechanical properties compared to other materials, such as PMMA–graphene composites and multilayered PMMA. The microstructural homogeneity of these materials is a key factor influencing their overall mechanical performance, as it contributes to their resistance to fracture and wear [112]. Furthermore, studies have shown that CAD/CAM materials, such as lithium disilicate and zirconia, possess high flexural strength, making them suitable for various restorative applications [16,214]. This strength is particularly important in areas subject to significant occlusal forces, where material failure could compromise the restoration’s integrity.

In addition to mechanical properties, the aesthetic qualities of CAD/CAM materials are also important to consider. The ability to achieve stable color and excellent aesthetics is one of the significant advantages of using CAD/CAM technology [215]. Materials such as lithium disilicate are known for their translucency and ability to mimic natural tooth structure, which enhances the overall appearance of restorations [16]. Moreover, advancements in material science have led to the development of hybrid composite resins that not only provide aesthetic benefits but also overcome limitations associated with traditional direct composite resins, such as polymerization shrinkage and defects. The standardized manufacturing processes of CAD/CAM materials ensure consistent color stability and surface finish, which are important for patient satisfaction [216].

The surface properties of CAD/CAM materials also play an important role in their clinical performance. Surface roughness is a critical factor that influences plaque accumulation and, consequently, the longevity of restorations. Studies have demonstrated that CAD/CAM materials can achieve surface roughness values below the threshold for plaque accumulation, thereby promoting better oral hygiene and reducing the risk of secondary caries [217]. Additionally, the polishing techniques applied to these materials can significantly affect their surface characteristics, with some methods having superior results in terms of smoothness and gloss [172]. The interaction of CAD/CAM materials with various dentifrices and bleaching agents also needs attention, as these can alter surface properties and potentially affect the longevity of restorations [218].

The adaptability of CAD/CAM materials to different clinical scenarios is another important aspect. For instance, the use of CAD/CAM technology allows for the fabrication of provisional restorations with enhanced mechanical properties compared to conventional materials. This capability is particularly beneficial in situations where immediate temporization is required, as it reduces chairside time and improves patient comfort [219]. Furthermore, the ability to repair CAD/CAM materials effectively is essential for maintaining restorations over time. Research conducted by Wiegand and co-workers has shown that thermocycling can enhance the bond strength of repaired CAD/CAM materials, indicating that these restorations can be effectively maintained in clinical practice [220].

The choice of CAD/CAM material also influences the clinical outcomes of restorations. For example, zirconia-based materials are preferred for their high strength and fracture resistance, making them suitable for posterior restorations [221,222]. Conversely, lithium disilicate is often preferred for anterior restorations due to its superior aesthetic properties [16]. The decision-making process concerning material selection is further influenced by factors such as the specific clinical situation, patient preferences, and the desired aesthetic outcomes [55].

**Figure 4:**
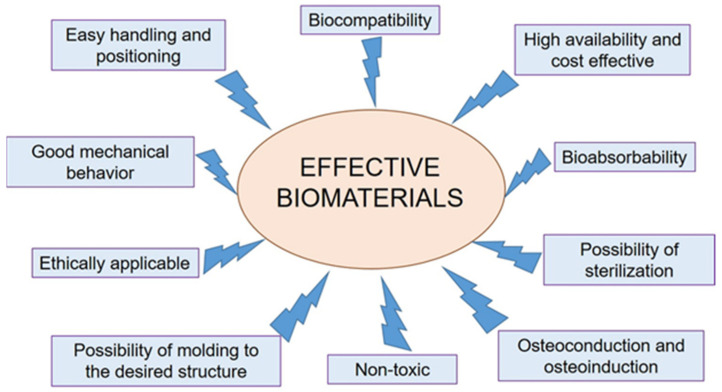
The main advantages of using CAD/CAM materials in dentistry applications.

Figure 4 presents the key characteristics that define the effective biomaterials used for CAD/CAM applications in dentistry.

Moreover, the evolution of CAD/CAM technology has led to the introduction of new materials that respond to the growing demand for metal-free restorations. These materials not only provide aesthetic benefits but also address concerns related to biocompatibility and patient comfort [47].

The increasing preference for metal-free restorations in dentistry can be attributed to both aesthetic and health considerations. Patients and practitioners are increasingly seeking materials that provide superior aesthetics without the drawbacks associated with metal restorations, such as visibility and potential allergic reactions. Zirconia and other ceramic materials have gained popularity due to their excellent aesthetic properties and biocompatibility, making them a favorable alternative to traditional metal–ceramic restorations [223,224,225]. Furthermore, advancements in CAD/CAM technology have facilitated the production of high-quality, metal-free restorations that meet the growing demand for aesthetic solutions [143,226]. Despite the advantages of metal-free materials, there are limitations to both CAD/CAM ceramics and metal blocks. While ceramics offer superior aesthetics, they can be more brittle and prone to fracture under occlusal forces compared to metals [227]. On the other hand, metal blocks, although durable, may not provide the same level of aesthetic appeal and can interfere with light transmission, affecting the overall appearance of restorations [143,161]. Additionally, the use of metal may pose challenges in terms of patient acceptance due to concerns over metal allergies and aesthetic preferences [228]. Therefore, while both material types have their place in restorative dentistry, the trend is clearly shifting towards metal-free options that align with contemporary patient expectations.

The ongoing research and development in this field continue to expand the range of available materials, allowing for clinicians to tailor their choices to meet individual patient needs effectively.

Table 3 presents a comparative analysis of the most used CAD/CAM materials, evaluating their mechanical and aesthetic properties. Specifically, it includes quantitative measures such as compressive strength, flexural strength, and fracture toughness, all expressed in megapascals (MPa). Additionally, qualitative attributes such as aesthetics, translucency, wear resistance, bonding to dentin, biocompatibility, longevity, and clinical indications are assessed. This comprehensive overview aids in the selection of appropriate materials for dental applications, emphasizing the balance between mechanical performance and aesthetic requirements.

## 5. Conclusions

The history of chairside CAD/CAM materials is characterized by a progressive shift from rudimentary systems to sophisticated technologies that improve the quality and efficiency of dental restorations. The continuous development of new materials and techniques promises to revolutionize this field further, making CAD/CAM an indispensable tool in contemporary dental practice. The transition from analog to digital workflows in chairside oral rehabilitation represents a significant advance in dental practice. Increased accuracy, improved patient outcomes, and increased efficiency are benefits that underline the importance of adopting digital technologies in modern dentistry. Therefore, the future of oral rehabilitation will be influenced by digital processes as future research continues to validate these approaches.

One of the main factors to consider when choosing the right material for CAD/CAM technology is its mechanical properties. Materials such as lithium disilicate and polycrystalline ceramics are preferred for their strength and durability, hence the longevity of the final dental restorations. The mechanical performance of CAD/CAM materials, including flexural strength and impact resistance, is essential to assure dentists that restorations can withstand the functional demands of the oral environment. The use of materials that combine the beneficial properties of ceramics and composite resins, such as polymer-infiltrated ceramic networks, improves the performance of chairside restorations. Aesthetic considerations also play a key role in material selection. The demand for aesthetic, metal-free restorations has led to the development of high-strength ceramics that can be used effectively with CAD/CAM technology. These materials not only meet the functional requirements but also offer excellent color stability and translucency, which are essential properties for natural-looking restorations.

The integration of CAD/CAM technology enables precise control over the design and fabrication process, allowing for dentists to create restorations that perfectly match the patient’s natural dentition. In addition, the efficiency of chairside CAD-CAM systems has a significant impact on clinical practice. The ability to fabricate restorations in a single visit reduces the number of appointments required, thereby improving patient comfort and satisfaction. Saving time is particularly beneficial in a busy dental practice, as it allows for increased patient flow and increased revenue potential for dental professionals. Overall, the use of CAD/CAM technology also minimizes the risk of cross-contamination associated with traditional laboratory processes, contributing to better infection control in dental practices. As advances in materials science and CAD/CAM technology continue to evolve, dental professionals have a wide range of options to effectively respond to the diverse needs of their patients.

## Figures and Tables

**Table 1:** Examples of commercially available CAD/CAM materials and their chemical composition.

CAD/CAM Material	Type of Material	Manufacturer	Chemical Composition
Vita Mark II	Feldspar-reinforced aluminosilicate glass	VITA Zahnfabrik, Bad Säckingen, Germany	>80% glass matrix <20% feldspathic particles
Vita Enamic	Hybrid ceramic (polymer-infiltrated ceramic network = PICN)	VITA Zahnfabrik, Bad Säckingen, Germany	86% feldspathic-based ceramic network 14% acrylate polymer network
Vita Suprinity	Zirconia-reinforced lithium silicate glass ceramic	VITA Zahnfabrik, Bad Säckingen, Germany	Glass–ceramic 10% zirconia (SiO_2_, Li_2_O, ZrO_2_, A1_2_O_3_, K_2_O, P_2_O_5_)
Vita CAD-Temp	Composite	VITA Zahnfabrik, Bad Säckingen, Germany	Acrylate polymer and microparticle filler
Vita In-Ceram Alumina	Aluminum oxide ceramic	VITA Zahnfabrik, Bad Säck-ingen, Germany	82% Al_2_O_3_, 12% La_2_O_3_, 4.5% SiO_2_, 0.8% CaO, and 0.7% other oxides
Cerasmart	Resin-based composite	GC, America, Alsip, IL, USA	71% silica and barium glass nanoparticles 29% resin matrix
GC-LRF	Leucite-reinforced ceramic	GC, America	Glass, oxides
Lava Ultimate	Resin-based nanoceramic (nanoparticulate pre-polymerized resin composite)	3M ESPE, Seefeld, Germany	80% nanoceramic (4–11 nm ZrO_2_, 20 nm SiO_2_, aggregated ZrO_2_/SiO_2_ cluster) 20% highly crosslinked polymer matrix resin
IPS e.max ZirCAD	Zirconium oxide ceramic	Ivoclar Vivadent, Schaan, Liechtenstein	Yttria-stabilized tetragonal zirconia polycrystals (3Y-TZP)
IPS e.max CAD	Lithium silicate ceramic	Ivoclar Vivadent, Schaan, Liechtenstein	Lithium disilicate crystals, LiO_2_, SiO_2_, K_2_O, P_2_O_5_, ZnO
IPS Empress CAD	Leucite-reinforced glass ceramic	Ivoclar Vivadent, Schaan, Liechtenstein	Leucite crystals, 60–65% SiO_2_, 16–20% Al_2_O_3_, 10–14% K_2_O, 3.5–6.5% Na_2_O, 0.5% other oxides, pigments
Polident PMMA	Polymethyl methacrylate	GlaxoSmithKline Plc, Mississauga, ON, Canda	Polymethyl methacrylate, pigments
PEEK-OPTIMA^TM^	Polyetheretherketone	Invibio Biomaterial Solutions, Thornton-Cleveleys, Lancashire, UK	Polyetheretherketone
KATANA™	Zirconia blocks	Kuraray Noritake Dental, Europe GmbH Philipp-Reis-Str. 4–65795, Hattersheim, Germany	Partially stabilized Yttria-stabilized zirconia (3Y-TZP); 90–95% ZrO_2_ with 5–10% Y_2_O_3_, small amounts of HfO_2_
BruxZir	Monolithic zirconia	Newport Beach, CA 92660, Glidewell	Partially stabilized 3Y-TZP zirconia; >90% ZrO_2_ with Y_2_O_3_, small amounts of alumina, and other trace oxides
CAMouflage	Ceramic polymer	Newport Beach, CA 92660, Glidewell	Polymethyl methacrylate with crosslinking agents, pigments, and fillers
BioTemps	Polymethyl methacrylate blocks	Newport Beach, CA 92660, Glidewell	Polymethyl methacrylate with crosslinking agents, pigments, and fillers
Obsidian	Monolithic glass ceramic	Newport Beach, CA 92660, Glidewell	Li_2_Si_2_O_5_ based glass–ceramic; >70% lithium disilicate, silica, additional metal oxides
Initial LRF Block	Leucite-reinforced ceramic	GC, America	Leucite-reinforced feldspathic porcelain, containing aluminosilicate glass, leucite crystals, glass-forming additives
Amber Mill	Lithium disilicate	HASS, Gangwon-do, Korea	Li_2_Si_2_O_5_, alkali metal oxides, coloring oxides
Tessera	Lithium disilicate	Dentsply Sirona, Bensheim, Germany	Multi-matrix resin-enriched with reinforced ceramic network (silica and zirconia)
Celtra Duo	Lithium silicate zirconia reinforced	Dentsply Sirona, Bensheim, Germany	Lithium silicate glass ceramic reinforced with ~10% ZrO_2_, Li_2_SiO_3_, Li_2_Si_2_O_5_, minor amounts of color oxides and nucleating agents
Paradigm MZ100	Bisphenol A-glycidyl methacrylate (Bis-GMA) composite	3M ESPE, Seefeld, Germany	Zirconia and silica fillers dispersed in a resin matrix (Bis-GMA, UDMA, TEGDMA); >80% inorganic filler content
Brilliant Crios	Bisphenol A-glycidyl methacrylate (Bis-GMA) composite	Coltene/Whaledent, Alstatten, Switzerland	~70% inorganic filler (barium glass, silica); UDMA resin matrix, filler particles are pre-polymerized
Grandio Blocks	Bisphenol A-glycidyl methacrylate (Bis-GMA) composite	Voco, Cuxhaven, Germany	~80% inorganic filler (nano-hybrid fillers, silanized ceramic); Bis-GMA, TEGDMA, UDMA resin matrix
LuxaCam composite	Bisphenol A-glycidyl methacrylate (Bis-GMA) composite	DMG Fabrik, Hamburg, Germany	A blend of di- and trimethacrylate resins, inorganic fillers (barium glass, silica), ~78% filler by weight
Tetric CAD	Bisphenol A-glycidyl methacrylate (Bis-GMA) composite	Ivoclar Vivadent, Schaan, Liechtenstein	~60% filler content, inorganic fillers (barium glass, silica); UDMA-based resin matrix
Shofu Block HC	Nanoceramic (fine particle silica (SiO_2_) hybrid polymer matrix)	Shofu, Kyoto, Japan	Methacrylate resins, filler particles consisting of dispersed silica
CEREC Zirconia e.max	Tetragonal zirconia	Dentsply Sirona, Bensheim, Germany	Zirconia core with an outer layer of Li_2_Si_2_O_5_ glass ceramic
Mazic Zir	Tetragonal zirconia	Vericom co., Gangwon-do, Korea	Partially stabilized yttria-stabilized zirconia (3Y-TZP); >90% ZrO_2_ with yttria, trace amounts of alumina, and other oxides
Mazic Pro	Polymethyl methacrylate	Vericom co., Gangwon-do, Korea	
LuxaCam Zircon HT Plus	Tetragonal zirconia	DMG Fabrik, Hamburg, Germany	Partially stabilized yttria-stabilized zirconia (3Y-TZP); >90% ZrO_2_ with yttria, trace amounts of alumina, and other oxides
LuxaCam PMMA	Polymethyl methacrylate	DMG Fabrik, Hamburg, Germany	Polymethyl methacrylate, crosslinking agents, pigments, stabilizers, and fillers
ArtBlock Temp	Polymethyl methacrylate	MERZ, Lütjenburg, Germany	Polymethyl methacrylate with crosslinking agents, pigments, and fillers

**Table 2:** CAD/CAM materials for restorative dentistry: optical properties, durability, and aesthetic considerations.

CAD/CAM Material	Optical Properties	Long-Term Aesthetic Durability	Monochromatic/Polychromatic
Feldspathic ceramics	High translucency, good opalescence, moderate light scattering	Moderate to good, susceptible to surface wear/staining	Predominantly monochromatic
Leucite-reinforced ceramics	Increased strength, moderate translucency, some opalescence	Moderate to good, moderate wear resistance	Predominantly monochromatic with stains/can be polychromatic
Lithium disilicates	High translucency (especially in low-opacity versions), good opalescence, moderate light diffusion	Good to excellent, high wear resistance, staining possible over time	Available in both monochromatic and polychromatic
Zirconia-reinforced lithium silicate	High strength and translucency (as compared to standard zirconia), good opalescence	Good to excellent, high wear and fracture resistance. good color stability	Available in both monochromatic and polychromatic
Zirconium oxide	Moderate to high opacity, low translucency (early generations), can be modified with layering to enhance translucency	Excellent, very high strength and fracture resistance but can appear less natural when full contour	Predominantly monochromatic (requires staining and layering)
Hybrid ceramics	Variable translucency (depending on filler load), moderate opalescence, moderate light reflection	Moderate to good, good flexibility, good color stability	Available in both monochromatic and polychromatic
Acrylic resin	Low to moderate translucency, low opalescence, prone to discoloration	Poor, susceptible to staining, wear, and fracture	Predominantly monochromatic (relatively poor color stability)

**Table 3:** The comparative properties of CAD/CAM materials concerning their bending strength, aesthetic qualities, and clinical applications.

Property	CAD/CAM Material						
	Feldspathic Ceramics	Leucite-Reinforced Ceramics	Lithium Disilicates	Zirconia-Reinforced Lithium Silicate	Zirconium Oxide	Hybrid Ceramics	Acrylic Resin
Bending Strength (MPa)	70–150	130–160	400–500	360–420	900–1200	80–120	50–70
Flexural Strength (MPa)	70–80	120–140	350–400	300–350	900–1200	90–150	70–90
Fracture Toughness (MPa√m)	1.5–2.5	2.0–2.5	2.0–3.0	3.0–4.0	6.0–10.0	1.5–2.0	1.0–2.0
Aesthetics	Excellent	Very Good	Excellent	Very Good	Moderate	Good	Moderate
Translucency	High	High	Very High	Moderate	Low	Good	Moderate
Wear Resistance	Good	Good	Excellent	Excellent	Excellent	Moderate	Low
Bonding to Dentin	Good	Good	Excellent	Excellent	Moderate	Good	Limited
Biocompatibility	High	High	High	High	High	Moderate	Good
Longevity	5–10 years	7–15 years	10–15 years	10–20 years	15–20 years	5–10 years	3–5 years
Indications	Veneers, anterior crowns	Anterior and posterior crowns	Inlays, onlays, full crowns	Posterior crowns, partial dentures	Full crowns, bridges	Low-load restorations, denture teeth	Temporary restorations, short-term

## Data Availability

No new data were created or analyzed in this study. Data sharing is not applicable to this article.
